# Progress in German Hospitals

**Published:** 1895-09-21

**Authors:** 


### HOSPITAL CONSTRUCTION.

### PROGRESS IN GERMAN HOSPITALS.

At a somewhat recent meeting of the Berlin Hygienic Society, a paper on "Recent Progress in Hospital Construction" was read by Herr Schmieden, a well-known architectural authority upon hospitals in Berlin. He commented upon the gradual and general substitution of stone for wood, and of the addition of a second storey in pavilion buildings, which at their first introduction consisted only of one; in England three or even four storeys being frequently adopted. The circular plan Herr Schmieden objected to as making a proper distribution of the buildings very difficult. He made special mention of the famous Hamburg Municipal Hospital with its fifty-eight pavilions, and the delightful arrangement by which the day rooms are provided with glass sides, which may be let down in warm weather, the rooms then becoming practically open balconies. The operation rooms had been built and furnished in accordance with the wishes of Dr. Schede, the principal surgeon, and were constructed only of iron, glass, and Dutch tiles, there being no woodwork whatever.

Amongst the most recently built hospitals, Herr Schmieden drew attention to the Kaiser Friedrich Hospital for Children in Berlin. Here very particular efforts had been made, in the portion devoted to the reception of diphtheria cases, to carry out an extensive system of isolation, the mild and serious cases being divided, and the convalescents being kept strictly away from either. Thus patients are received in one of two wards, according to the severity of the disease, and from thence are moved into the convalescent ward without entering any other part of the building. The resident medical men and nurses live in the diphtheria pavilion, never entering any other department. Herr Schmieden attached much importance to these precautions, instancing cases where infection had been conveyed through the nurses working in the different wards all living together.

Amongst other novelties in recent hospital planning, that in the Policlinico Umberto, in Rome, was worthy of notice, where in the operating theatre, for the more complete antiseptic performance of operations, these are carried on in a kind of glass cage, through which the surgeon can be seen, but not heard, by the surrounding students.

Dealing with the cost of hospital buildings, Herr Schmieden spoke of England, and remarked that the cost was much higher relatively there than on the Continent, where in Germany 4,000 marks per bed is the usual rule, whilst in England 8,000 is nearer the average. The conclusion finally arrived at by the lecturer was a wise one, for, as he pertinently observed, the best results in hospital construction were only to be arrived at through the hearty co-operation and collaboration of architects and medical men.

